# High coverage fluid-phase floating lipid bilayers supported by ω-thiolipid self-assembled monolayers

**DOI:** 10.1098/rsif.2014.0447

**Published:** 2014-09-06

**Authors:** Arwel V. Hughes, Stephen A. Holt, Emma Daulton, Andrei Soliakov, Timothy R. Charlton, Steven J. Roser, Jeremy H. Lakey

**Affiliations:** 1 ISIS Pulsed Neutron Source, Rutherford Appleton Laboratory, Harwell Science and Innovation Campus, Harwell OX11 0QX, UK; 2 Bragg Institute, Australian Nuclear Science and Technology Organisation, Locked Bag 2001, Kirrawee DC 2001, New South Wales 2232, Australia; 3 Institute for Cell and Molecular Biosciences, The Medical School, Newcastle University, Newcastle upon Tyne NE2 4HH, UK; 4 Department of Chemistry, University of Bath, Claverton Down, Bath BA2 7AY, UK

**Keywords:** floating supported bilayer, neutron reflection, thiolipid, self-assembled monolayer, gold, magnetic contrast

## Abstract

Large area lipid bilayers, on solid surfaces, are useful in physical studies of biological membranes. It is advantageous to minimize the interactions of these bilayers with the substrate and this can be achieved via the formation of a floating supported bilayer (FSB) upon either a surface bound phospholipid bilayer or monolayer. The FSB's independence is enabled by the continuous water layer (greater than 15 Å) that remains between the two. However, previous FSBs have had limited stability and low density. Here, we demonstrate by surface plasmon resonance and neutron reflectivity, the formation of a complete self-assembled monolayer (SAM) on gold surfaces by a synthetic phosphatidylcholine bearing a thiol group at the end of one fatty acyl chain. Furthermore, a very dense FSB (more than 96%) of saturated phosphatidylcholine can be formed on this SAM by sequential Langmuir–Blodgett and Langmuir–Schaefer procedures. Neutron reflectivity used both isotopic and magnetic contrast to enhance the accuracy of the data fits. This system offers the means to study transmembrane proteins, membrane potential effects (using the gold as an electrode) and even model bacterial outer membranes. Using unsaturated phosphatidylcholines, which have previously failed to form stable FSBs, we achieved a coverage of 73%.

## 1. Introduction

Phospholipid bilayers deposited on solid substrates are very useful systems for studying the structures, behaviours and interactions of biological membranes [1]. Localizing a membrane to a surface enables it to be studied by the plethora of techniques available to surface science. Additionally, in contrast to free floating vesicles, a substrate imposes a known orientation relative to the laboratory plane, and so studies where the orientation of components is important (such as emulating natural asymmetric membranes) become easier. Similarly, membranes on surfaces could form the basis of new sensor technologies potentially aiding diverse fields such as drug delivery studies or disease diagnostics [2,3].

A number of approaches have been proposed, including Langmuir–Blodgett (LB) techniques, spontaneous vesicle fusion onto either uncoated or pre-modified surfaces, and self-assembled tethered bilayers [4,5]. However, the common problem with these approaches is that, the mobility of the component lipids can be severely limited owing to their interactions with the substrate surface. A natural biomembrane is a dynamic, fluid system, where the component molecules have considerable translational freedom, and this fluidity is central to the behaviour of the system. In a deposited bilayer, however, the loss of translational freedom means that the bilayer is, at best, only a poor approximation of the real system and suitable for probing only the simplest interactions.

Early attempts to preserve fluidity in supported bilayers were centred on single lipid bilayers on Si/SiO_2_ and quartz surfaces [6]. It was shown that under conditions of sufficient hydration, a thin water layer (less than 10 Å) forms between the bilayer and the hydrophilic surface of the substrate, which potentially acts as a cushion insulating the membrane from substrate effects. However, the thermal fluctuations within the membrane were suppressed leading to the absence of the *pβ*′ phase of saturated lipids [7] (which relies on significant out of plane motions of the lipids) suggesting a strong coupling between the substrate and the phospholipids. These single lipid bilayer systems were used in studies of peripheral membrane proteins. However, incorporation of integral membrane proteins was found to be problematic since the cushioning water layer was too thin to prevent large proteins impinging on the substrate surface [8].

Attempts to overcome the immobilization problem have largely involved pre-coating the substrates prior to bilayer deposition. So for example, Wong *et al.* [9] fused lipid vesicles of dimyristoyl phosphatidylcholine (DMPC) onto layers of hydrated polyethylenimine, and while there was evidence of increased lipid mobility in these systems, the structure of the final assembly was found to be highly dependent on the preparation method. Other thin hydrophilic polymer film materials have also been used such as dextran [10], but using 600–800 Å thick dextran films, stable monolayers of DMPC could only be formed with the addition of significant amounts of cholesterol (20 mol%). Wagner & Tamm [11] incorporated molecules of dimyristoyl phosphatidylethanolamine (DMPE), polyethyleneglycol and a triethoxysilane group into the lower leaflet of dioleoyl phosphatidylcholine (DOPC) bilayers. The authors demonstrated good lateral mobility of membrane proteins which are not free to diffuse in single supported membranes. However, the composition of the lower leaflet leads to membranes that do not truly mimic natural systems. This restriction is likely also to be true of bilayers directly tethered to surfaces using thiolipids with a hydrophilic spacer which have nevertheless enabled the reconstitution of large integral membrane proteins [2–4,12,13].

It has been known for many years that multilayers of lipids deposited on solid supports do retain the freedom necessary to act as realistic model systems. In such films, generally consisting of hundreds of bilayer units, the individual bilayers remain separated by gaps of up to 30 Å, and evidence for a weak interaction of these units with the substrate has been obtained by a close inspection of their phase behaviour, which closely mimics that of bilayers in vesicles [14]. However, rather than being submerged, the films are examined in a humid atmosphere, making the study of membrane–solute interactions difficult.

The large separation between the bilayers in these stacks is caused by Helfrich repulsion [15], analogous to the force that maintains the separation between the bilayers in multilamellar vesicles (MLVs). In MLVs, adjacent bilayers associate with each other through electrostatic and Van der Waals forces but are prevented from sticking together by a balancing repulsive entropic pressure. That is, if the bilayers were to adhere directly to each other, then their natural fluctuations would be suppressed, leading to a decrease in their overall entropy. This manifests as a repulsion between the bilayers resulting in an extensive layer of water. The key to forming useable supported bilayers that are not influenced by friction with the substrate is therefore to mimic the balance of forces seen in multilamellar vesicles, but in a fully submerged system.

The first such system was the so-called floating lipid bilayer described by Fragneto *et al.* [16]. In their model, a double bilayer was produced in a highly controlled way using a combination of LB and Langmuir–Schaeffer (LS) techniques. In these methods, ordered monolayers of the lipids are first fabricated at the air–water interface, and then transferred to the substrate in a layer by layer fashion by slowly bringing the substrate into contact with the monolayer (the difference between the Blodgett and Schaeffer methods is in the orientation of the substrate, with the substrate being perpendicular to the air–water interface or parallel, respectively) [17]. By using a sequence of four such transfers (3 × LB + 1 × LS), they were able to fabricate fully submerged double bilayer structures. The systems were fully characterized by neutron reflectivity and it was shown that the upper bilayer, while remaining strongly associated with the lower layers, was separated from them by the large distance expected from Helfrich effects. The bilayer showed the expected changes in thickness across the main phase transition, and the intermediate *P_*β*′_* ‘ripple’ phase’ was also detected, indicating a low constraining effect of the substrate in these systems.

Despite this promise, fabrication of a double bilayer system was only possible for a subset of lipids, specifically those in the gel phase above room temperature, in this case, saturated phosphatidylcholines longer than C16 such as dipalmitoyl-sn-glycero-3-phosphocholine (DPPC). The four layers are deposited sequentially, but in the case of shorter (or unsaturated) lipids, the bottom bilayer was seen to desorb during the deposition of the third layer, and hence the double bilayer system could not be formed [18]. The limited range of lipids which can be used to form the double bilayer therefore limits its usefulness, as native systems invariably contain a significant portion of unsaturated lipids and these cannot be mimicked by the double bilayer.

Hughes *et al.* [18] replaced the lowermost layer of the system with a grafted self-assembled monolayer (SAM) of octadecyltrichlorosilane, improving the stability of the lower supporting layers during the fabrication of the floating bilayer. Using this approach, it was possible to deposit DMPC which has a shorter chain length (C14) and is therefore in the fluid phase at room temperature. The OTS/DMPC system was again studied by neutron reflection, and it was shown that the lipids within the upper layer have the same area per molecule (APM) and thickness as in DMPC multilamellar vesicles, suggesting that the lipids organize according to their own steric demands rather than according to any constraining influence of the substrate [19]. The ripple transition was also detected in this system.

Experimental difficulties with this system remain, that is, the OTS layer quality varies significantly from sample to sample, thus limiting the usefulness of this approach. A subsequent improvement was to replace the two-layer lower support with a single chemically grafted phosphatidylcholine layer [20]. The support for the floating bilayer is then a robust and reusable support, which can be fully characterized in the absence of the floating membrane, greatly aiding subsequent characterization of the floating membrane itself. However, despite extensive optimization, full coverage of the grafted OTS–SAM is never achieved, with a coverage of 70–80% of the substrate being the norm [21]. This then leads to a commensurate reduced coverage of the subsequent bilayer which can cause problems in studies of agents that penetrate the membrane, i.e. it is difficult to distinguish between a peptide that truly penetrates the bilayer and one that simply locates in holes and imperfections.

**Figure 1.**
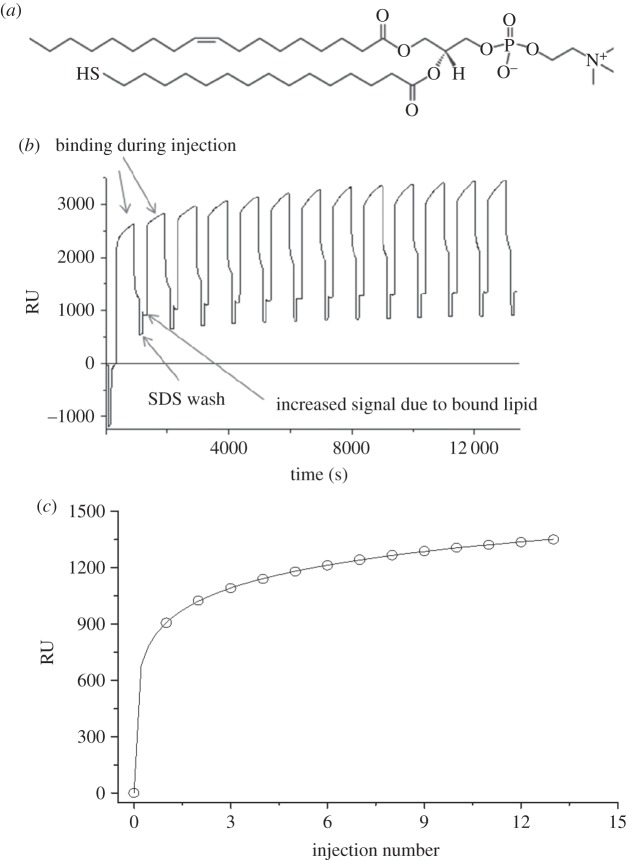
(*a*) ω-Thiolipid (1-oleoyl-2-(16-thiopalmitoyl)-sn-glycero-3-phosphocholine); (*b*) surface plasmon resonance data. Bare gold chips were mounted in a Biacore X-100 and thiolipid 1 mg ml^−1^ in 1% octylglucopyranoside, 50 mM Tris pH 8.0, 10 mM TCEP was injected at a flow rate of 5 μl min^−1^ for 10 min, after which the surface was washed with 1% v/v SDS for 100 s before repeating the cycle 12 times. The amount of thiolipid bound after each injection was determined by the increase in resonance units each wash; these successive values were plotted against injection number (*c*) and fitted to estimate the maximum saturation coverage.

In this paper, we describe a new approach to fabricating FSBs, where the silane-grafted phosphatidylcholine on silicon is replaced by a thiol-grafted phosphatidylcholine (figure 1*a*) on a gold surface. Kycia *et al.* [22] assembled FSB on gold electrodes covered with a 1-thio-β-d-glucose SAM and visualized them using AFM, whereas thiolipids have been used previously to create very dense SAMs on gold. Crucially, the thiolipid used here differs from those previously used [2,12,23,24] in that the thiol group is at the ω-end of the palmitoyl chain leading to a dense SAM with 100% orientation of the choline headgroup towards the solvent. We show that the coverage of the ω-thiolipid SAM is much greater than that typically seen for the silane SAMs, and similarly the coverage of the resulting FSB is greatly improved. We describe an FSB of fluid phase DPPC, produced on this ω-thiolipid surface, where neutron reflection data suggest that coverage approaches 100%. The possibility of placing a magnetic layer beneath the gold allows additional contrasts to be generated by the use of polarized neutrons [25]. There is also a significant advantage for membrane potential dependent or electrochemical studies of membranes, because a defect free (highly insulating) membrane is essential and the gold can act as an electrode. Finally, we show that the dense thio-phospholipid SAM allows the fabrication of FSBs containing unsaturated lipids, in this case 1-palmitoyl-2-oleoyl-*sn*-glycero-3-phosphocholine (POPC), overcoming a limitation of previous FSBs.

## 2. Experimental set-up

### 2.1. Sputtering of alloy/gold surfaces

A binder layer was first sputtered onto the silicon to improve the gold adhesion. Rather than the typical chromium or titanium binder layer, a soft magnetic alloy, permalloy (Ni : Fe 4 : 1), was used to enable magnetic contrast neutron reflectometry (MCNR) [25]. The layers were sputtered at the NIST Center for Nanoscale Science and Technology, Gaithersburg, MD, USA, in a Denton Discovery 550 sputtering chamber. The magnetic (≈120 Å) and gold (≈150 Å) layers were deposited sequentially in the same chamber.

### 2.2. Fabrication of the ω-thiolipid self-assembled monolayer

ω-Thiolipid (1-oleoyl-2-(16-thiopalmitoyl)-sn-glycero-3-phosphocholine; Avanti Polar Lipids; figure 1*a*) was dissolved at 1 mg ml^−1^ in chloroform/methanol (4 : 1), dried as a film in a glass tube and resuspended in 1% octylglucopyranoside (OG) (Sigma), 50 mM Tris pH = 8.0 (OG buffer). Immediately prior to use, tris (2-carboxyethyl)phosphine HCl (TCEP) was added to a final concentration of 1 mM. The gold surfaces were cleaned by sonication in 2% Hellmanex solution (Hellma GmbH), rinsed and then sonicated in 1% sodium dodecyl sulfate (SDS) solution. The cleaned surfaces were immersed in the ω-thiolipid/OG solution for 2 h at 50°C. The surfaces were removed from the lipid solution, cleaned again in SDS and re-immersed in the lipid solution for a further 2 h. Finally, the surfaces were removed from the coating solution, washed with SDS, rinsed with ultrapure water UPW (Millipore; 18.2 mΩ cm^−1^) and dried under nitrogen.

### 2.3. Deposition of floating bilayers

The LB/LS depositions were carried out using a purpose-built LB trough (Nima Technology, Coventry, UK). The trough has a large, deep dipping well to accommodate the horizontally oriented silicon crystals during the Schaeffer transfer. The trough was cleaned and filled with UPW. Either DPPC or POPC (Avanti polar lipids) was dissolved in chloroform at approximately 1 mg ml^−1^, and 300 µl was spread onto the cleaned water surface. After waiting 15 min to allow the monolayer to equilibrate, the monolayer was compressed at 50 cm^2^ min^−1^ to 30 mN m^−1^. For the first, LB transfer, the compression was carried out with the substrate already immersed in the trough. After reaching target pressure, the substrate was withdrawn upwards through the monolayer at 5 mm min^−1^, with the trough running in constant pressure mode.

For LS transfers, a clean neutron reflection cell was placed in the trough before it was filled. After filling the trough, the monolayer was spread and compressed to the required pressure, and the substrate mounted horizontally on the dipper such that its gold face was parallel to the water surface. The tilt of the substrate was adjusted using a motor-controlled levelling stage, such that the substrate and the water were exactly parallel. The substrate was then pushed through the interface at a speed of 5 mm min^−1^ and then sealed in the neutron cell while still underwater, as described previously [16,18,20].

### 2.4. Surface plasmon resonance analysis of thiolipid layer

Bare gold Biacore AU-chips (GE Healthcare) cleaned with 2% Hellmanex were used in a Biacore X-100 with a running buffer of phosphate-buffered saline pH 7.4, 0.05% v/v Tween (polysorbate) 20. The thiolipid prepared as above (1 mg ml^−1^ in OG buffer) was injected at a flow rate of 5 µl min^−1^ for 10 min, after which the surface was washed with 1% v/v SDS for 100 s before repeating the cycle 12 times. The amount of thiolipid bound after each injection was determined by the increase in resonance units (1 RU = 10^−4^ degree shift in the angle of reflectivity minimum) when running buffer replaced the SDS containing buffer used in each wash. The flow system used in the Biacore does not achieve saturation levels possible by static incubation, so successive injection values were plotted against injection number and fitted to the extended Langmuir equation (equation (3.1)) to estimate the saturation coverage.

### 2.5. Magnetic contrast neutron reflectometry

Reflectometry experiments were carried out on three beam lines, Polref and Crisp at ISIS, Oxfordshire, UK and Platypus at the Opal Research Reactor, Sydney, Australia. All three instruments operate in time-of-flight mode where a white beam of neutrons is incident on the sample; see the electronic supplementary material, table S1 for instrument parameters and a description of data collection and reduction. All the beam lines were equipped with polarizers that transport a single neutron spin state to the sample, placed in an external magnetic field, such that the magnetic moments of the permalloy layer are aligned either parallel or anti-parallel to the neutron beam polarization. The direction of magnetization at the sample is fixed, and upstream spin flippers are used to alternate the polarization of the incoming neutron beam. Data were collected at a number of neutron incident angles onto the sample with the perpendicular momentum transfer, *q_z_*_,_ typically ranging from 0.008 to 0.3 Å^−1^ where, *q_z_* = (4π/*λ*)sin*θ*, *θ* is the incident angle and *λ* is the neutron wavelength.

In an X-ray or neutron reflection experiment, the reflected intensity is measured as a function of incident angle. For a single interface, the specularly reflected intensity decays as a function of the fourth power of *q_z_*. Where distinct layers exist on a substrate, reflection will occur from each of the interfaces between the layers, and interference between the multiple reflections modifies the decay of the reflected intensity to produce interference fringes. A specular reflectivity curve essentially probes the neutron refractive index normal to the substrate surface, or more correctly, the square modulus of its Fourier transform [26]. Because the Fresnel coefficients are squared, the phase information is effectively lost in reflection data (the so-called phase problem), and the consequence of this is that the reflectivity data cannot be directly transformed into a unique density profile. Instead, any given reflectivity curve can be obtained from a set of scattering length density profiles.

To counteract this problem of non-uniqueness, it is usual to measure samples in neutron reflectivity at a series of ‘contrasts’. The basic idea is to measure a series of reflectivity datasets, which share the same structural parameters but differ in the scattering length densities (SLDs) of the bulk phases or one or more of the layers. Then, by simultaneously analysing all the datasets, the number of potential numerical solutions is greatly reduced and, in the limit of enough datasets, can tend to a unique solution [26]. Conventionally, for samples at the solid/water interface, this contrast variation is achieved by simply replacing the bulk water phase by one or more mixtures of H_2_O and D_2_O, because the neutron scattering lengths of deuterium and hydrogen differ greatly.

More recently, a novel method of introducing contrast variation has been developed, using spin-polarized neutrons and their differential interactions with magnetizable layers [25]. The effective SLD of magnetizable layers varies according to the spin direction of the atoms in the layer relative to that of the neutrons in the incoming beam. Therefore, by introducing a magnetizable under-layer into the substrate and applying a magnetic field at the sample, either parallel or anti-parallel to the neutron spins in the incoming beam, the effective SLD of this layer changes according to the neutron spin direction providing additional contrasts without altering the biological sample. In this study, both methods of contrast variation were used, with the samples measured against both D_2_O and H_2_O, with each measured at both spin states to give four simultaneous contrasts per sample.

### 2.6. Data analysis strategy

The neutron reflection data were analysed using a conventional ‘layer model’, where the interface is subdivided into a series of layers. Each layer is defined by a thickness, the interfacial roughness between it and the adjacent layer, and the average neutron refractive index within the region of the interface corresponding to that layer. The reflectivities were calculated from the layer parameters using the recursive Parratt formalism [27]. As is usual in reflection studies of amphiphilic layers, we divide the thiolipid layer (and later the bilayer) into the hydrophobic ‘tail’ region and the hydrophilic ‘head’ regions to model the reflection data. For the case of the bilayers, a further layer is introduced between the two back to back chains to represent the central methyl region, which is known to have a different density to the remainder of the hydrophobic core of the bilayer, and so each bilayer was modelled as five layers in total. The bilayers were considered to be symmetrical, with both leaflets described by the same set of parameters.

The layers were parametrized using known lipid volumes [28] (electronic supplementary material, table S2) as described in the text. The models were fit to the data using a Bayesian approach, whereby the *n*-dimensional posterior distribution function was examined using a nested sampling algorithm [29,30]. The log-likelihood was defined in terms of the usual chi-squared statistic [31], with all contrasts analysed simultaneously and the sum of chi-squared over all contrasts defining the likelihood distribution. Uniform priors were employed. For each parameter, the samples were histogrammed, and the maximum of each distribution was taken as the best-fit parameter value, whereas the uncertainties were taken as the shortest (iteratively obtained) 95% CI of each distribution [31].

## 3. Results and discussion

### 3.1. Thiolipid self-assembled monolayer formation and characterization

The static contact angle of water on the bare gold was less than 10° and following SAM formation increased to 54 ± 0.2° (electronic supplementary material, figure S1), which is consistent with a previous study of phosphatidylcholines chemisorbed onto gold surfaces [26]. The SAM assembly was investigated using surface plasmon resonance (SPR). Figure 1*b* shows the SPR over the course of 13 × 600 s injections of the ω-thiolipid/OG solution. Between each injection, the cells were rinsed with 1% SDS for 100 s, and then running buffer. During each injection, a rapid increase in SPR was seen and when the cell was flushed with SDS to remove the non-bound lipids and detergents a decrease in signal was observed. However, after each wash, a net increase in signal remained, indicating surface-bound material resistant to SDS washes. This residual signal increases rapidly over the first couple of injections before tending towards saturation (figure 1*c*). We estimated the maximum saturation value by fitting the data to an extended Langmuir model such that3.1
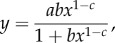
where *x* is the injection number, *a*, *b* and *c* are coefficients. This process provided an estimate for *y*_max_ of 2680 ± 344 RU (*r*^2^ = 0.999) equal to a 0.268° shift in the SPR minimum. Assuming a refractive index of the lipid of 1.45, a 1° shift on the Biacore X-100 is equivalent to a layer thickness of 7.6 nm. Thus, our 0.268° shift indicates a lipid thickness of 2.04 nm, reasonable for a single adsorbed layer of lipid.

**Figure 2.**
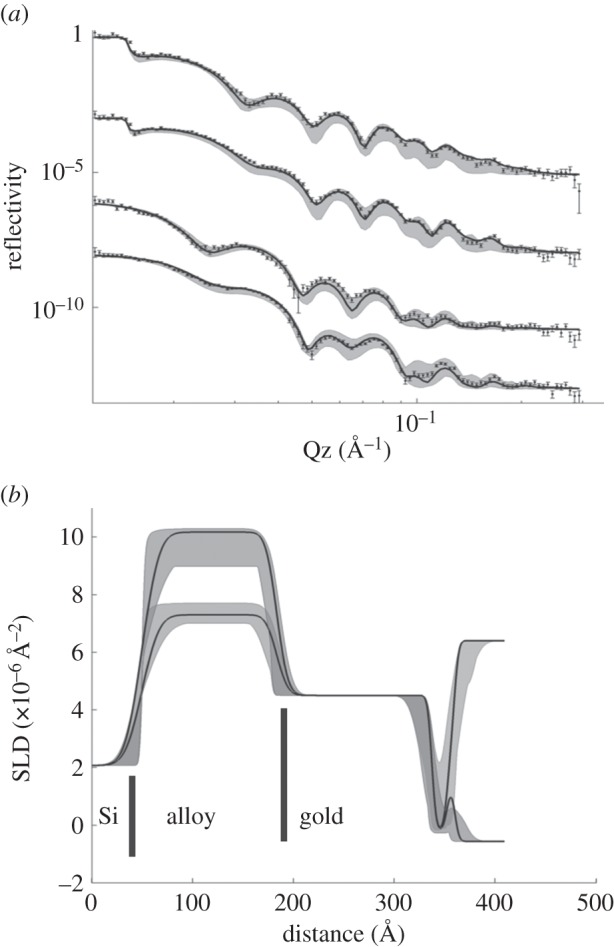
(*a*) Neutron reflectivity data for the SAM coated surface from four magnetic and solution contrasts, and simultaneous fit using the lipid volumes from the electronic supplementary material table S2, and the model described in the text. The curves are offset vertically for clarity and are, from top to bottom; D_2_O up spin, D_2_O downspin, H_2_O up spin, H_2_O downspin. The solid curves through each dataset are the best fits obtained from the Bayesian analysis, whereas the lighter grey shading on each curve represents the ‘spread’ of profiles obtained between the 95% CIs given in table 2. (*b*) Scattering length density (SLD) profiles corresponding to the fits in (*a*). The profiles differ in two regions, between distances of 100 and 150 Å the up spin data fits to a higher SLD, and the down spin to a lower SLD for the permalloy layer and at the maximum distance the D_2_O data fits to the higher value for the solution SLD and the H_2_O fits the lower solution SLD value. The black lines represent the mean profiles, and the shaded regions the range of SLD profiles corresponding to the shaded regions of uncertainty on the reflectivity data in (*a*).

The ω-thiolipid SAMs were then analysed by MCNR. The samples were measured against both D_2_O and H_2_O, at both ‘up–up’ (parallel) and ‘down–down’ (anti-parallel) spin polarizations, leading to four contrasts in total as described in the previous section (figure 2*a* for example).

The data were analysed using standard layer models, with the layer parameters calculated from known lipid volumes. In such models, each layer is defined by three parameters: a thickness, a roughness and an SLD. For systems where the layers are strongly associated with distinct molecular regions, information about the chemical composition of each layer can be used to parametrize the models such that the number of fitting parameters is reduced. The SLD, *ρ*, of a given layer is related to the molecular structure of its components, because3.2
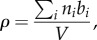
where *n_i_* is the number of atoms of the *i*th atom, *b_i_* is the bound coherent scattering length of the *i*th atom and *V* is the molecular volume. So if the composition and molecular volume of a given layer are known, then the SLD can in principle be calculated exactly. For a given layer, its thickness (*τ*) will then be governed by the projected interfacial APM (*A*),3.3
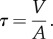
In submerged lipid layers, the headgroups are extensively hydrated, and so these water molecules must be accounted for in the calculation of the SLD and thickness. Assuming a mean number of water molecules per headgroup (*n*_W_) as a fitting parameter, the volumes and elemental composition of the headgroup layers are then adjusted to account for the extra contribution of the hydrating water. In addition to the SLDs and thicknesses, a roughness parameter is required for each layer. To simplify the models and to reduce the number of required fitting parameters, we assume a contiguous roughness for both hydrophobic and hydrophilic layers, which is a reasonable assumption at the resolution afforded by the limited q-range of neutron reflection.

The overall SLD profile calculated from this model will be that of a complete, defect-free layer. It is possible that defects (in the form of holes) could exist in any SAM, and these would be filled with water. To allow for this possibility, we introduce a ‘coverage parameter’, essentially expressing the volume fraction of solvent in the form of defects in the film. Then, the overall SLD of each layer is given by a scaled sum of the layer SLD and that of the bulk water, so that3.4
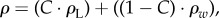
where *C* is the fitted volume fraction of the layer, *ρ*_L_ is the SLD of the given layer, and *ρ*_*w*_ is the SLD of the solvent. In other words, for a complete layer, *C* will tend towards unity and decrease in value as the coverage of the layer decreases. A lipid layer can then be defined in terms of just four fitting parameters—the projected interfacial area (*A*), the mean number of hydrating water molecules in the headgroup regions (*n_W_*), the roughness (*r*) and the coverage (*C*). This approach of using known lipid volumes to reduce the number of fitting parameters required is conventionally used in parametrizing models for analysing reflection data from amphiphilic layers [32]

The best fits are shown in figure 2, and the best-fit parameter values in tables 1 and 2. The fits suggest that the thiolipid SAM is essentially complete and defect-free, with the fits converging to a coverage of more than 99%. The APM of the lipid is around 51.5 Å^2^. The number of waters per headgroup is not well resolved however, with a mean value of around nine water molecules per lipid, but with a large spread of values around this mean. The SAMs were stable for the duration of the experiments but deteriorated when kept dry for 12 months and needed to be reassembled after first cleaning the gold surfaces (electronic supplementary material, table S3).

**Table 1.** Best-fit values (maxima of the parameter probability histograms from the Bayesian analysis) applied to the ω-thiolipid SAM; DPPC FSB and POPC FSBs. These values correspond to the fits shown in figures 2, 3 and 5. The bracketed ranges are the shortest 95% CIs of each histogram. APM, area per molecule; SLD, neutron scattering length density; WPH, water molecules per lipid headgroup.

sample parameter	fitted values (95% CI)	fitted values (95% CI)	fitted values (95% CI)	prior limits
	SAM only	DPPC	POPC	
substrate roughness (Å)	6.66 (4.0, 9.47)	5.15 (4.45, 10.427)	10.06 (2.98, 19.90)	4, 20
alloy thick (Å)	133.76 (130.46, 137.24)	136.21 (134.75, 137.53)	134.62 (132.92, 136.17)	100, 200
alloy SLD ↑↑ (10^−6^ Å^−2^)	7.34 (7.01, 7.58)	7.36 (7.28, 7.45)	7.12 (7.01, 7.23)	6.0, 12
alloy SLD ↓↓ (10^−6^ Å^−2^)	9.88 (9.57, 10.2)	9.95 (9.87, 10.02)	10.1 (9.93, 10.16)	6, 13
alloy rough (Å)	5.19 (3.80, 9.13)	9.09 (7.10, 9.96)	7.08 (5.38, 9.42)	2, 10
gold thick (Å)	152.31 (148.29, 156.28)	152.98 (151.23, 154.67)	150.75 (148.93, 153)	100, 200
gold rough (Å)	6.52 (4.23, 9.06)	4.13 (2.14, 5.92)	7.2138 (5.17, 9.82)	2, 10
thiolipid APM (Å^2^)	52.37 (40.66, 61.40)	57.93 (51.20, 64.34)	53.66 (48.34, 59.10)	40, 100
thiolipid; WPH	8.30 (1.02, 10.43)	1.49 (1.01, 15.12)	7.99 (2.14, 12.04)	1, 30
thiolipid coverage	0.98 (0.84, 0.999)	0.996 (0.912, 1.0)	0.98 (0.96, 1.00)	0.5, 1
central water thick (Å)	—	16.2 (7.42, 19.27)	17.37 (12.18, 22.91)	1, 25
bilayer APM (Å^2^)	—	63.72 (59.14, 67.66)	85.008 (62.489, 103.35)	48, 90
bilayer WPH	—	3.42 (1.01, 14.87)	12.75 (8.39, 19.60)	1, 30
bilayer rough (Å)	—	3.7 (3.00, 5.34)	6.91 (6.12, 9.49)	3, 15
bilayer coverage	—	0.992 (0.934, 0.999)	0.76 (0.60, 0.90)	0.5, 1

**Table 2.** Comparison of bilayer parameters determined from the FSBs by neutron reflection compared with values determined by X-ray diffraction from lipid multilayer stacks.

	DPPC		POPC	
	this work	XRD [14]	this work	XRD [33]
APM	63.56 (58.64, 68.05)	64	84.92 (59.78, 109.93)	68.3
*n_w_*	11.24 (1.01, 17.50)	8.6	17.67 (6.35, 29.1)	9.4
coverage	0.97 (0.91, 0.99)	—	0.76 (0.61, 0.91)	—

### 3.2. Floating supported bilayer fabrication and characterization

**Figure 3.**
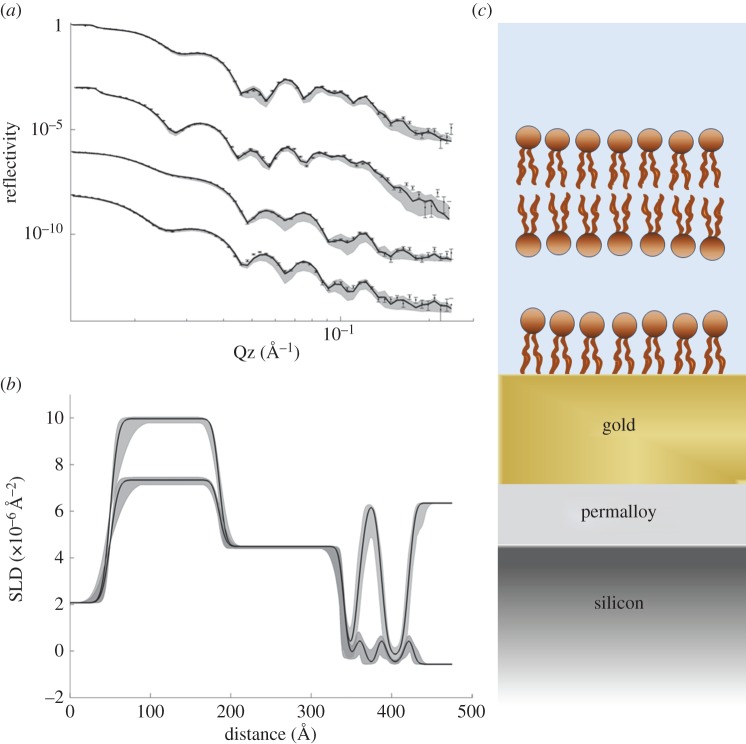
(*a*) Neutron reflectivity data for a DPPC floating bilayer at 50°C from four magnetic and solution contrasts and (*b*) corresponding SLD profiles. The structure is as shown schematically in (*c*), and the order of the reflectivity contrasts is as in figure 2. The pronounced central water region is clearly visible in the SLD profile and its SLD changes according to whether the solvent is D_2_O or H_2_O. The SLD of the organic regions however do not change appreciably with water contrast indicating a high coverage. (Online version in colour.)

**Figure 4.**
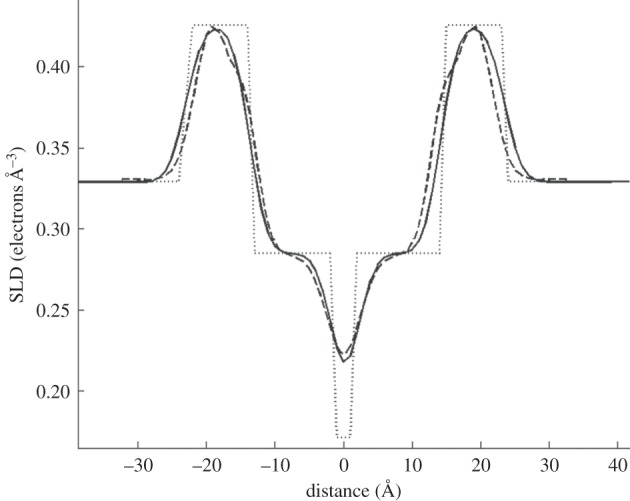
Comparison of the SLD profile for the DPPC bilayer from the box model in this work (solid line) with that obtained from X-ray diffraction (dashed line, from [33]). The dotted line shows the positions of the unroughened slabs which make up the box model. The roughness applied to the box model to obtain the solid line was 2.5 Å—lower than the best-fit value but within the range of uncertainty.

Bilayers of both a saturated (DPPC) and unsaturated (POPC) lipid were fabricated. The decrease in trough area as a function of dipper height during the initial LB transfers was linear (electronic supplementary material, figure S2) for both lipids. Accurate transfer ratios cannot be calculated, because the three bare silicon faces of the block also pass though the monolayer. The deposited FSBs were analysed by MCNR as for the SAM alone, and these data for DPPC at 50°C (i.e. in the physiologically relevant ‘fluid’ phase) are shown in figure 3, and for POPC at room temperature in figure 4.

The data analysis strategy for the FSB samples is similar to that employed for the SAM alone, in that the bilayers are subdivided into layers, the thickness and SLDs of each of the layers calculated from known lipid volumes. We again treat the head and tail regions separately, and for the bilayer we introduce a further layer at the bilayer centre containing the methyl groups of each leaflet. The packing of the central methyl region is known to often differ from the remainder of the alkyl region, and is indeed strongly resolved in X-ray diffraction measurements of membranes [33]. The bilayers are treated as symmetric, and therefore a single APM parameter is used throughout each bilayer. Similarly, a single roughness parameter is used for all the interfaces in each membrane, the same mean number of water molecules per headgroup is assumed for each leaflet, and one coverage parameter is used to account for defects in the membranes. The only additional parameter required is the thickness of the central water layer, which means that the FSB data are modelled by 16 parameters in each case (table 1).

It is known from investigations of FSBs deposited on silane SAMs that the surface density, or coverage, of the underlying support controls the eventual coverage of the floating bilayer [21]. With silane SAMs, surface coverage was rarely more than 75%, and the coverage of resulting DPPC bilayers matched that of the SAM. This is also the case here, with the DPPC bilayer on the 99% SAM showing a coverage of ≈97%, the highest yet reported for any FSB.

Between the bilayer and the SAM is a ≈17 Å water layer. Reported separations between FSB and underlying substrates vary with the chain length of the bilayer lipids, and their phase. The water thickness in the gel phase decreased with increasing chain length, from 22 Å for DPPC (C16), to 19 Å for DSPC (C18), to as low as 15 Å for C20 PC, reflecting a slight increase in bending modulus with chain length [34]. Upon heating to the fluid phase, the separation increased to a large maximum around the main melting transition, and decreased again to lower values in the fluid phase about 2–3 Å smaller than the gel phase, and so the fluid phase values measured here agree with previous reports. Similarly, for a DSPC bilayer on a grafted silane-phosphatidylcholine SAM [20], the gel phase water layer thickness is 17.4 ± 0.9 Å. Our value for the mean number of water molecules per headgroup (approx. 7.6) is also very close to theirs (8.6), but as we discuss later, our value is not very well defined. We are currently measuring the effects of different variables such as buffer composition which, in addition to SAM structure and those mentioned above, are likely to affect the water layer thickness [17]. The APM of DPPC in the fluid phase has been considered previously, and was reviewed by Nagle & Tristram-Nagle [14]. They found a very large spread in reported values obtained by different techniques, but they were able to converge on an APM of 64 Å^2^ at 50°C, in good agreement with the 65 Å^2^ from our analysis.

The SLD profile from the box model fits (transformed into X-ray units), can be compared with a published SLD profile for fluid-phase DPPC (obtained from X-ray diffraction from in stacked bilayers [33]) as shown in figure 4. As can be seen, there is excellent correspondence between the two. Some of the finer details of the headgroup structure are not captured by the box models—the ‘peaks’ which correspond to the headgroup's electron density are asymmetric in shape in the diffraction data, but are symmetric Gaussians in the box model case—but over the limited *q_z_* range available to neutrons there is insufficient resolution for the reflectivity to be affected by these small differences. It has been shown that box models fail at the far higher *q*-values accessible from X-ray reflectivity measurements [30], and more complex models are required in which the headgroups are further subdivided into smaller fragments. For X-ray studies of lipid monolayers, layer models diverge from models of higher spatial resolution at a *q_z_* of about 0.5 Å^−1^ [30]. However, the *q_z_* ranges of all the data considered here are well within this limit, and so box models are an appropriate level of approximation.

**Figure 5.**
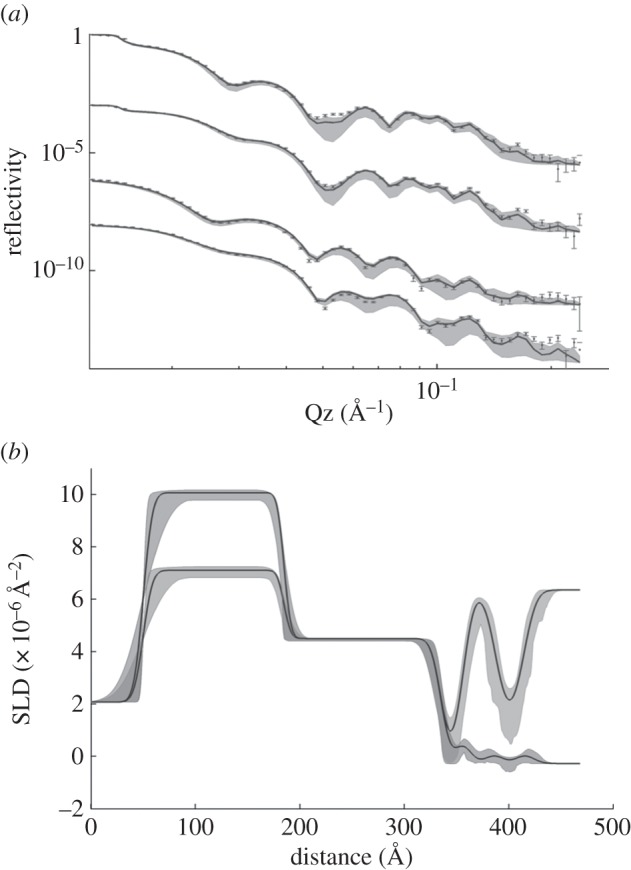
(*a*) Neutron reflectivity data for a POPC floating bilayer from four magnetic and solution contrasts and (*b*) corresponding best-fit SLD profiles. See figure 2 for details.

A disadvantage of FSBs supported on non-grafted lower layers is the inability to fabricate FSBs from unsaturated lipids. Following the transfer of the initial two LB layers, the third ‘peels off’ the previous layers thus increasing in the area occupied by the film at the air–water interface [18]. The grafted lower layers prevent this, and allow the fabrication of unsaturated films as is clear from the transfer shown in the electronic supplementary material, figure S2. The neutron reflection data (figure 5) confirm that a bilayer has formed from monounsaturated lipids (POPC), and it is clear merely by inspection of figure 5*b* that the pronounced, relatively thick hydration layer characteristic of floating bilayers is also present.

Accurate analysis of this unsaturated lipid sample is problematic, as the values of many of the fitted parameters at convergence vary widely. The thickness of the central water layer, for example, has a mean value of 19.7 Å, which again is consistent with other FSBs, but could range from 18.7 to 23 Å. Similarly, the number of water molecules per headgroup and the bilayer roughness are both poorly defined. The coverage of the bilayer is reduced relative to DPPC, suggesting around 72% bilayer surface coverage, with a large spread in values ranging from 54 to 85%. Kucerka *et al*. [35] report that unsaturated lipids occupy larger interfacial areas than saturated lipids of similar chain lengths. For POPC, they report an APM of 68.3 ± 1.5 Å^2^, lower than our mean value of 80 Å^2^, although it does lie within the range of uncertainty.

It would seem that obtaining an accurate structural determination of the bilayers from reflectivity data therefore benefits from the ability to make high-quality samples of good coverage, but for neutron scattering there is one other experimental aspect that is available to improve the determination of the parameters of interest. Against D_2_O, the headgroups are essentially invisible against the sharp interface between the alkyl chains and the solvent and even in H_2_O their contribution is weak, appearing as rather small ‘peaks’ in the SLD profiles. Partially deuterated samples of POPC and DPPC are available commercially, including those with deuteration of the headgroups only and studies of partially deuterated membranes are currently underway.

As for the lower coverage of POPC, it is known from studies of LB films generally that the deposition conditions (pressure, speed, subphase temperature) can have a large effect on the quality of the resulting films [36]. The POPC films transferred during this work were fabricated using the ‘standard’ conditions for saturated lipids as a guide, and there is no guarantee that these will be optimal for unsaturated lipids. However, even without optimization, coverage of (up to) 76% for an unsaturated floating bilayer is satisfactory for many investigations where coverage is less important, such as optical microscopy studies.

## 4. Conclusion

The simplicity, stability and completeness of the layers created here provide significant improvement in the methods available to fabricate floating bilayers. The addition of a gold layer enables the use of SPR and the application of electrical potentials while the ability to add a magnetic layer greatly improves the accuracy of the neutron reflectivity measurements. There is no possibility of mixing between the SAM and FSB, whereas the latter can be formed from two separate monolayers allowing for an asymmetric FSB to be created. In the case of saturated lipids, the reduction in defects and increase in density may reduce flip–flop and maintain any asymmetry applied during FSB formation.

## Supplementary Material

Supplementary data

## Supplementary Material

Data
