# Supplementary material for: High coverage fluid-phase floating lipid bilayers supported by ω-thiolipid self-assembled monolayers
Source: J R Soc Interface. 2014 Sep 6;11(98):20140447. doi: 10.1098/rsif.2014.0447 (PMC4233693; doi:10.1098/rsif.2014.0447)

High Coverage Fluid-Phase Floating Lipid Bilayers supported by ω-Thiolipid SAMs.

Arwel V. Hughes, Stephen A. Holt, Emma Daulton, Andrei Soliakov, Timothy R. Charlton, Steven J. Roser and Jeremy H. Lakey.

Data Collection and Reduction:

Neutron Reflection (NR) data was collected on the three beamlines outlined in Table S1. Each instrument was operated such that a rectangular area of *ca* 25 x 60 mm^2 of the 40 x 100 mm gold coated silicon disks was illuminated by the neutron beam. This was kept constant by adjustment of the collimation slits for all incident angles. A 'straight-through' run where the beamline is configured in the zero angle position and the silicon substrate placed at the sample position such that the neutron beam passes through the bulk silicon for the same path length as a data run, was collected for each set of slit configurations used in the experiments. On Platypus, where the area detector used has a stringent count rate limitation, the beam onto the detector was attenuated by an oscillating slit for the straight-through runs. The data reduction was then carried out following a very similar procedure for each instrument. The data runs were divided through by data from the appropriate straight-through run. The runs from different angles on one instrument were then 'stitched' together using an overlapping Q-region. If required, the dataset was then scaled so that below the critical edge the reflectivity was unity. For datasets without a critical edge, for example H_2O subphase, the scaling factor from the previous D_2O run was applied. Once this procedure was completed all datasets were treated in exactly the same manner when it came to data fitting and modelling.

Table S1. Basic data collection for the Neutron Reflectomers used in this work.

Instrument	Location	Wavelength range (Å)	Incident Angles (°)	Q Range ($Å^{-1}$)
Polref	TS2, ISIS	1.5 – 17.0	0.5, 2.0	0.009 – 0.24
CRISP	TS1, ISIS	0.5 – 6.5	0.35, 0.8, 1.8	0.012 – 0.65
Platypus	OPAL, ANSTO	3.0 - 16.0	0.45, 1.4, 4.2	0.007 - 0.3

High Coverage Fluid-Phase Floating Lipid Bilayers supported by ω-Thiolipid SAMs.

Arwel V. Hughes, Stephen A. Holt, Emma Daulton, Andrei Soliakov, Timothy R. Charlton, Steven J. Roser and Jeremy H. Lakey.

Figure S1 – Static contact angles of a freshly cleaned gold surface (upper), and after exposure to the thiolipid solution (lower).

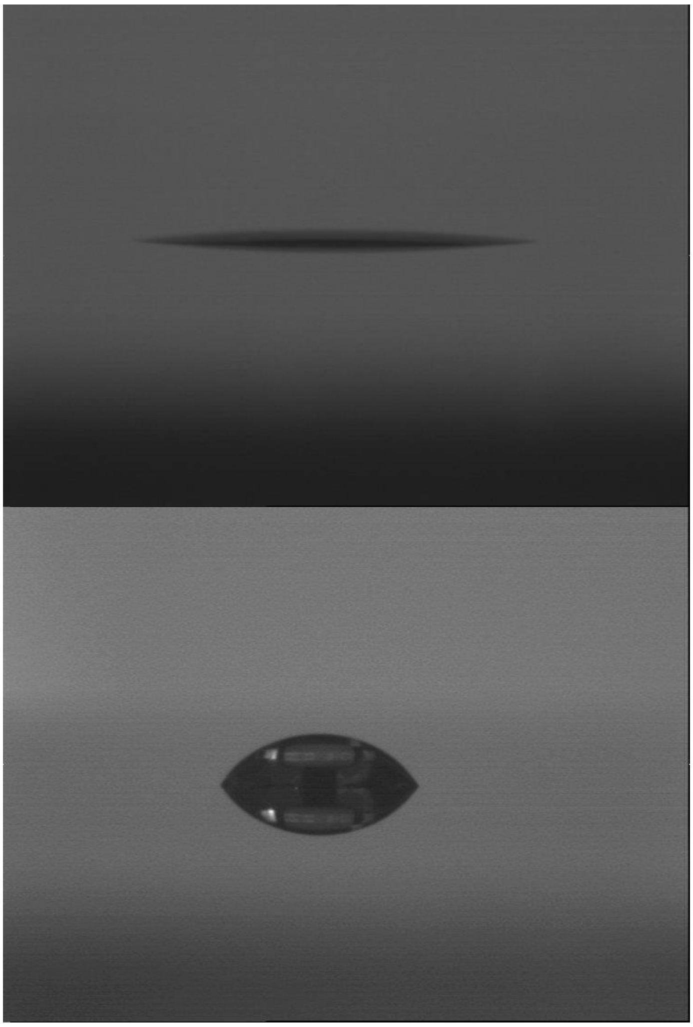

High Coverage Fluid-Phase Floating Lipid Bilayers supported by ω-Thiolipid SAMs.

Arwel V. Hughes, Stephen A. Holt, Emma Daulton, Andrei Soliakov, Timothy R. Charlton, Steven J. Roser and Jeremy H. Lakey.

Table S2 Molecular volumes of lipid component groups, from Armen *et al*. (1)

Group	Volume/Å^3
CH_3	52.7 ± 1.2
CH_2	28.1 ± 0.1
C=C	45.0 ± 1.6
CARB	39.0 ± 1.4
GLY	68.8 ± 9.9
PHOS	53.7 ± 2.4
CHOL	120.4 ± 5.0

1. Armen, R. S., O. D. Uitto, and S. E. Feller. 1998. Phospholipid component volumes: Determination and application to bilayer structure calculations. Biophysical Journal 75:734-744.

High Coverage Fluid-Phase Floating Lipid Bilayers supported by ω-Thiolipid SAMs.

Arwel V. Hughes, Stephen A. Holt, Emma Daulton, Andrei Soliakov, Timothy R. Charlton, Steven J. Roser and Jeremy H. Lakey.

Figure S2 Trough area as a function of time during the Langmuir-Blodgett transfer of the two lipids, a) DPPC & b) h-POPC, onto the SAM coated surfaces. The linear decrease in area during the LB dips (defined by the arrows) are consistent with uniform transfer.

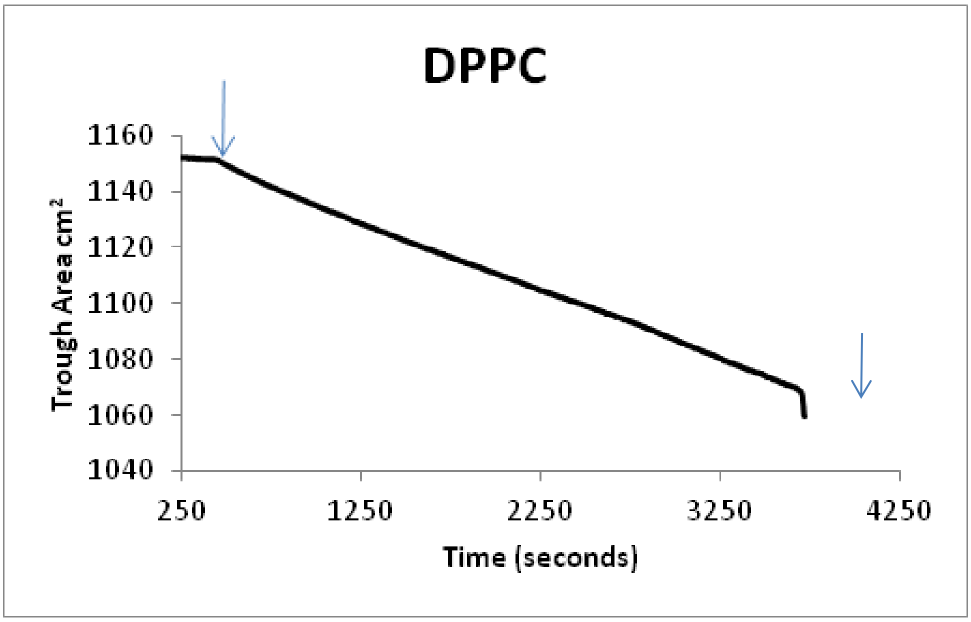

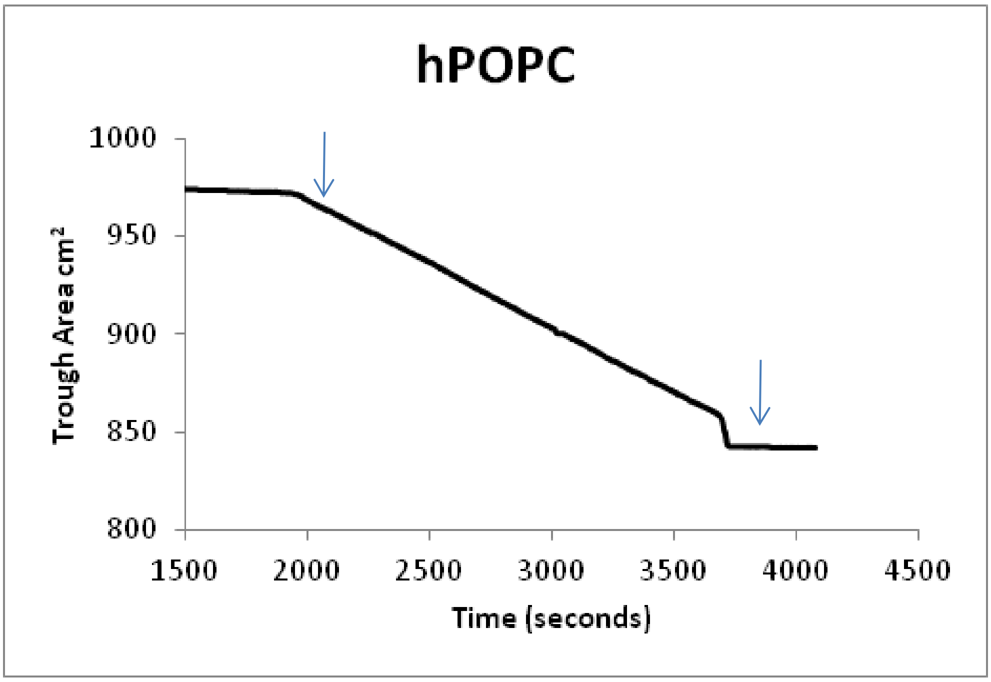

Supplement: Supplementary data [file rsif20140447supp1.pdf]
